# Long-term Stability in Endoscopic Brow Lift: A Systematic Review and Meta-Analysis of the Literature

**DOI:** 10.1093/asj/sjae225

**Published:** 2024-11-14

**Authors:** Serhat Şibar, Asiye Uğraş Dikmen, Ayhan Işık Erdal

## Abstract

Long-term stability and recurrent ptosis are among the most debated topics in endoscopic brow lifts. Although there are many publications on endoscopic brow lifts, more research is needed on long-term brow elevation and stability. In this systematic review we aimed to evaluate the amount of elevation and stability achieved by endoscopic brow lifts in the long term. To evaluate the long-term outcomes of endoscopic brow lifts, the databases PubMed, Web of Science, Scopus, and Google Scholar were searched with the keywords “endoscopic brow lift,” “endoscopic forehead lift,” “forehead lift,” “foreheadplasty,” “brow lift,” “endoscopic brow fixation,” and “brow fixation.” Studies published between September 1994 and May 2024, including isolated or combined endoscopic brow lift surgeries, were included. In total, 5324 articles were screened, and 85 full texts were reviewed. Of these studies, 12 (14.1%) were found suitable for systematic review and meta-analysis. Brow elevation values were evaluated separately by medial, central, and lateral parts. The pooled effect sizes for medial, central, and lateral brow elevations were found to be 3.25 mm (2.44-4.06), 3.86 mm (2.93-4.8), and 4.35 mm (3.06-5.64), respectively. This study is the first meta-analysis to present the average elevation values that can be achieved in the long term by endoscopic brow lifts. These data guide a better understanding of patient candidates and endoscopic brow lift technique. Sharing more objective data over the long term about different fixation methods will contribute to a better understanding of the criteria related to indications for this surgery.

**Level of Evidence: 3 (Therapeutic):**

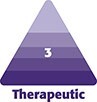

The eyebrows are one of the first regions affected by the aging process of the face. The reduction in skin elasticity, the atrophy of subcutaneous fat tissue, and the imbalance in the muscles responsible for eyebrow depression and elevation result in brow ptosis.^1-4^ Knize's anatomical studies have shown that the lateral part of the eyebrow undergoes ptosis more rapidly than the medial part. This has been associated with reduced frontal muscle activity laterally and the presence of fat compartments located over the temporal fascia.^5^ Lateral hooding frequently observed in patients can also be explained by this mechanism.

Endoscopic brow lift is a method that corrects the changes observed in the medial and lateral parts of the eyebrow. First described by Isse and Vasconez, this technique has advantages such as being minimally invasive, causing less tissue trauma, preserving neurovascular structures, and having a short recovery time.^6-8^ With these benefits, it is not surprising that brow lift procedures have gained popularity in recent years. According to the Aesthetic Plastic Surgery National Databank Statistics 2023, brow lift procedures have shown an 18% increase from 2022 to 2023 and a significant 54.7% increase compared to 2019.^9^ This reflects both the growing demand and advancements in aesthetic surgery techniques. The success of the technique depends on adequate stabilization following extensive periosteal dissection. Various fixation methods such as the cortical tunnel technique, Endotine, percutaneous screws, plates and screws, Kirschner wires, tissue adhesives, bolster dressings, fascial fusion (suturing of the superficial temporal fascia to the deep temporal fascia), galeapexy, and MiTek anchor systems have been described.^4,10-17^ Furthermore, some studies suggest that fixation may not be necessary for brow elevations less than 4 mm.^18^ Recurrent ptosis and unpredictable relapses encountered in patients over the long term are among the most frequently criticized and debated topics regarding endoscopic brow lifts today.^1,19-23^ The existence of numerous fixation materials for stabilization supports the ongoing search in this area. Despite nearly 30 years having passed since the technique was first introduced, the number of studies reporting on evaluation of long-term effectiveness with quantitative data in the literature is quite limited.^21,24-26^ We aimed to systematically compile studies evaluating the long-term effectiveness of endoscopic brow lifts quantitatively and present the findings of the meta-analysis.

## METHODS

### Study Protocol

A comprehensive search was conducted in the PubMed (National Institutes of Health, Bethesda, MD), Web of Science (Clarivate, Philadelphia, PA), Scopus (Elsevier, Amsterdam, the Netherlands), and Google Scholar (Alphabet, Inc., Mountain View, CA) databases from the date of the first publications related to endoscopic brow lifts up until May 2024, with the keywords “endoscopic brow lift,” “endoscopic forehead lift,” “forehead lift,” “foreheadplasty,” “brow lift,” “endoscopic brow fixation,” and “brow fixation.” The search was kept broad with the selected keywords, resulting in 5324 articles. After excluding duplicates and articles that were not appropriate based on the title and abstract reviews, the full texts of 85 remaining articles were reviewed. These articles were evaluated for eligibility for systematic review and meta-analysis based on inclusion and exclusion criteria.

The inclusion criteria were as follows: Randomized controlled, cohort, prospective, or retrospective case series studies; studies in which the classic surgical technique (anterior scalp with or without temporal region incisions, subperiosteal dissection plane) was applied for endoscopic brow lift; studies specifying scalp fixation methods; studies with a follow-up period of at least 1 year; studies in which brow elevation was measured quantitatively in millimeters with specific anatomical landmarks (such as medial canthus, midpupil, lateral canthus) in the periorbital region with the eyes open; and full-text clinical studies published in English.

The exclusion criteria were as follows: case reports, commentaries, letters to the editor, personal experience, or book chapters; studies applying methods other than the classic surgical technique (pretrichial or midforehead endoscopic approaches, subgaleal or subcutaneous dissection planes); cadaver or experimental animal studies; studies in which botulinum toxin or fillers were applied simultaneously with the endoscopic brow lift or in the early postoperative period; studies in which brow elevation was not quantitatively measured; studies in which evaluation of brow elevation was reported in percentage or ratios, without measurements in millimeters; studies in which the fixation method was not specified; studies with a follow-up period of less than 1 year; studies in which measurements were with vectors other than vertical (oblique); and studies not published in English.

**Figure 1.**
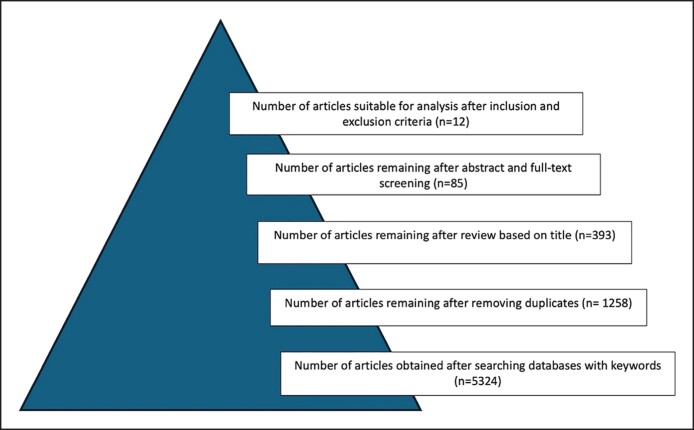
Flowchart of the systematic review in the study.

Studies were evaluated by 2 independent authors (S.S., A.I.E.), and disagreements were resolved by consensus. A total of 12 studies were found suitable for systematic review and meta-analysis (Figure 1). Oxford Center evidence-based medicine criteria were applied to assess the quality of evidence in the included studies.^27^

### Statistical Analysis

A series of meta-analyses were conducted with random effects models to estimate the average medial, lateral, and central brow elevation values. These analyses were estimated with the DerSimonian-Laird method, which is appropriate for addressing heterogeneity between studies. Various heterogeneity measures were performed to assess and interpret the variability between the included studies. The Q-statistic was applied to determine whether the observed differences in effect sizes across studies were greater than what would be expected by chance. A significant Q-statistic indicates the presence of heterogeneity, whereas the I^2^ statistic measures how much of the total variation is due to heterogeneity rather than chance. Values of 25%, 50%, and 75% represent low, moderate, and high heterogeneity, respectively. To assess the potential impact of publication bias on meta-analytic results, the rank correlation test (Kendall's τ) and Egger test were performed. These tests were employed to detect asymmetries in funnel plots that might indicate publication bias. The meta-analyses were conducted with the JASP software (version 0.18.3).

### Model Estimates and Tests

For medial averages, the pooled effect size had an intercept of 3.25 (SE: 0.412; 95% CI: 2.442-4.057). The omnibus test of model coefficients was significant (Q = 62.211, df = 1, *P* < .001), and the residual heterogeneity test also yielded significant results (Q = 388.388, df = 8, *P* < .001). The I^2^ value of 97.73% within the 95% CI of 94.005%-99.299% indicated substantial true heterogeneity.

For central averages, the pooled effect size had an intercept of 3.86 (SE: 0.479; 95% CI: 2.925-4.803). The omnibus test of model coefficients was significant (Q = 65.047, df = 1, *P* < .001), and the residual heterogeneity test was also significant (Q = 175.971, df = 10, *P* < .001). The I^2^ value of 93.07% within a 95% CI (83.951%-97.416%) indicated substantial true heterogeneity.

**Figure 2.**
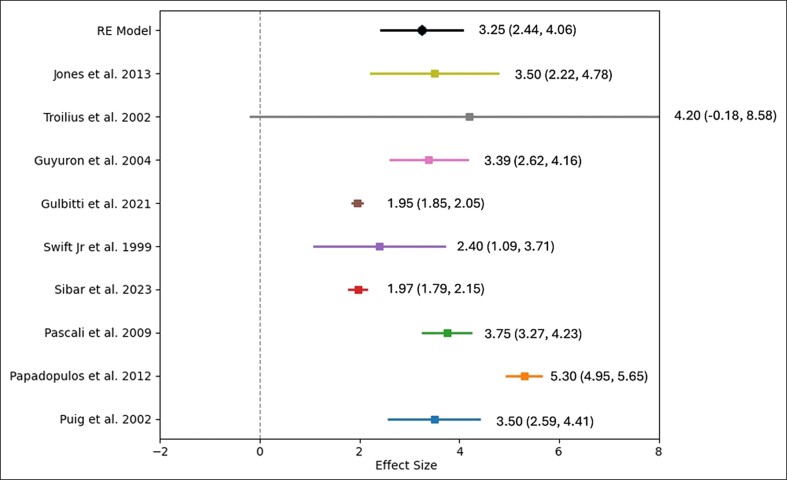
Forest plot graphic for the medial brow average elevation values.

**Figure 3.**
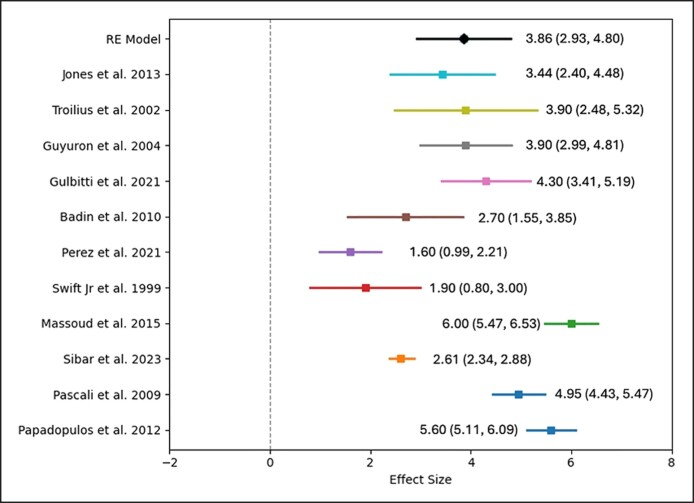
Forest plot graphic for the central brow average elevation values.

**Figure 4.**
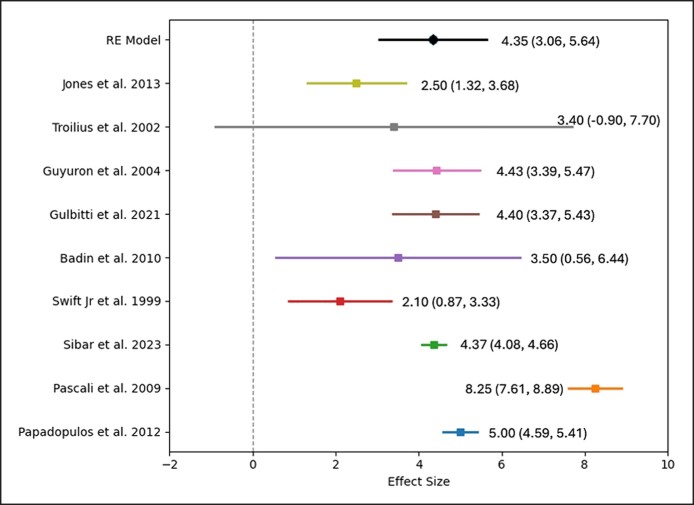
Forest plot graphic for the lateral brow average elevation values.

For lateral averages, the pooled effect size had an intercept of 4.35 (SE: 0.657; 95% CI: 3.062-5.636). The omnibus test of model coefficients was significant (Q = 43.875, df = 1, *P* < .001), and the residual heterogeneity test was also significant (Q = 155.204, df = 8, *P* < .001). The I^2^ value of 96.169% within the 95% CI (90.273%-98.873%) again indicated substantial true heterogeneity (Figures 2-4).

### Publication Bias

**Figure 5.**
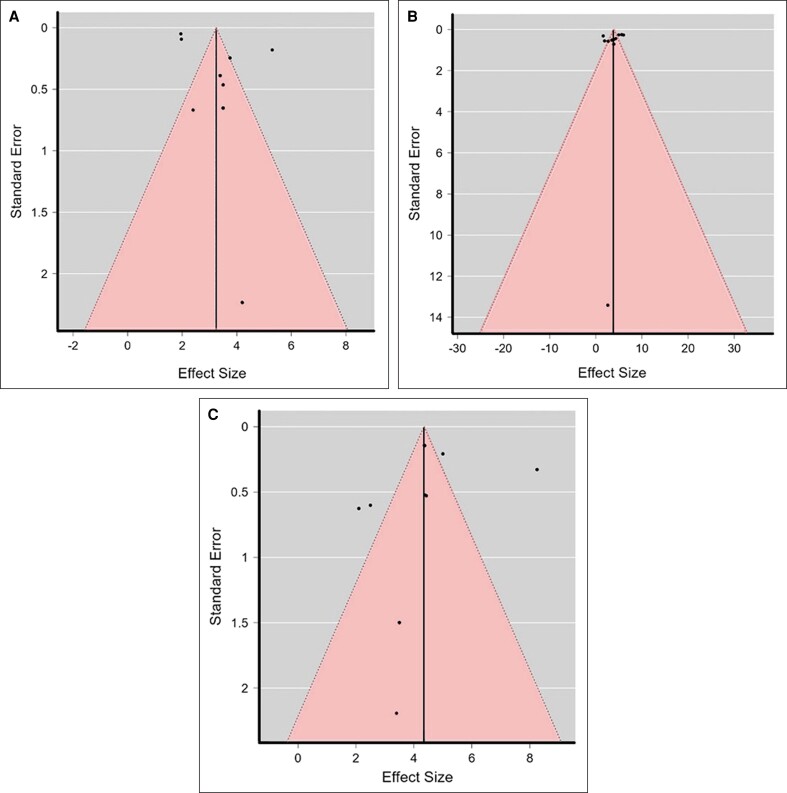
Funnel plots of the effect sizes of the included studies for medial (A), central (B), and lateral (C) averages.

The tests for publication bias, including Kendall's rank correlation and Egger's regression tests, revealed no significant asymmetry in the funnel plots for the estimates of medial, central, or lateral averages (Kendall's τ = 0.111, *P* = .761; Egger's z = −0.323, *P* = .747; Kendall's τ = −0.273, *P* = .283; Egger's z = −0.222, *P* = .824; Kendall's τ = 0.111, *P* = .761; Egger's z = −0.323, *P* = .747, respectively) (Figure 5). This indicates that no bias was applied in the selection of studies for the systematic review and meta-analysis.

## RESULTS

### General Characteristics and Demographic Findings of the Studies

It was determined that 12 of the 85 articles (14.1%) that underwent full-text review evaluated long-term stability with objective data. The majority of these articles were retrospective in nature (91.7%, *n* = 11), with only 1 study (8.3%) being prospective. Half of the studies (*n* = 6) were published in or before 2010, and the other half (*n* = 6) were published after 2010. The level of evidence for 11 studies (91.7%) was determined to be level 4, and 1 study (8.3%) was classified as level 3. Eight studies (66.7%) specified the type of computer software programs utilized for objective measurements.

The total number of cases with numerical measurements following endoscopic brow lift was 478. The gender distribution of the cases was specified in 9 studies (75%), with the majority being female (92.4%, *n* = 316). The average age of the cases was reported in 10 studies, ranging between 43.7 and 63 years. Average follow-up durations were reported in 9 studies (75%), with follow-up periods ranging between 1 and 2 years in 5 studies and exceeding 2 years in 4 studies. In 3 studies, the follow-up periods were noted to be at least 12 months, but exact data were not provided (Table 1).^4,15,18,24,25,28-34^

**Table 1.** General Characteristics and Demographic Data of the Included Studies

Author	Type of study	Year	Country	Level of evidence	Software	Number of cases measured	Gender	Mean age (min-max)	Mean follow-up (months)
Swift et al^26^	Retrospective	1999	USA	4	NA	20	19F, 1M	54 (30-73)	15.9 months
Puig and LaFerriere^27^	Retrospective	2002	USA	4	NA	38	31F, 7M	53 (NA)	16.9 months
Troilius^17^	Retrospective	2004	Sweden	4	Mirror	60	NA	NA	Minimum 12 months
Guyuron et al^28^	Prospective	2005	USA	4	Adobe Photoshop	48	46F, 2M	43.7 (23-63)	Minimum 12 months
Pascali et al^29^	Retrospective	2009	Italy	4	NA	30	26F, 4M	50 (38-70)	18 months
Badin et al^30^	Retrospective	2010	Brazil	4	Mirror	44	43F, 1M	50 (33-69)	Minimum 12 months
Papadopulos et al^14^	Retrospective	2012	Germany	4	JASC	52	52F	63 (48-71)	66 months
Jones and Lo^22^	Retrospective	2013	England	4	Adobe Photoshop	31	NA	NA	64.8 months
Massoud and Aboelatta^31^	Retrospective	2015	Egypt	4	NA	31	31F	Group 1: 47.6 (NA) Group 2: 51.3 (NA)	14.1 months
Perez et al^4^	Retrospective	2021	USA	4	Emotrics	33	26F, 9M	62.6 (40-85)	15.8 months
Gülbitti et al^23^	Retrospective	2022	Netherlands	3	Iconico	47	NA	55 (35-72)	72 months
Şibar et al^32^	Retrospective	2023	Türkiye	4	Image J	44	42F, 2M	51.2 (35-67)	24.7 months

F, female; M, male; NA, not available.

In 5 studies, it was found that some cases had undergone upper blepharoplasty along with endoscopic brow lift, and no information on upper blepharoplasty was available in 5 studies. In 1 study, none of the cases underwent upper blepharoplasty, and in another study, it was noted that upper blepharoplasty was performed if needed after the endoscopic brow lift.

### Modification of Depressor Muscle Groups

The studies varied in their approaches to the corrugator, procerus, and orbicularis oculi muscle groups. In most studies myotomy was performed of these muscles, whereas others reported no intervention (Table 2).

**Table 2.** Other Characteristics of the Included Studies

Author	Number of cases with upper blepharoplasty	Depressor muscle (corrugator or procerus) modification	Medial brow fixation	Lateral brow fixation	Medial brow average elevation amount (mm) ± SD	Central brow average elevation amount (mm) ± SD	Lateral brow average elevation amount (mm) ± SD
Swift et al^26^	NA	Yes	No	Cortical screw + stapler	2.4 ± 3.0	1.9 ± 2.5	2.1 ± 2.8
Puig and LaFerriere^27^	NA	Yes	Cortical tunnel + suture (2/0 Prolene)	Fascial fusion (2/0 Prolene)	3.5 ± NA	NA	3.2 ± NA
Troilius^17^	30 cases	Yes	No (only ligamentous release)	No (only ligamentous release)	4.2 ± NA	3.9 ± NA	3.4 ± NA
Guyuron et al^28^	NA	Yes	No	Fascial fusion (3/0 PDS)	3.39 ± 2.7	3.9 ± 3.23	4.43 ± 3.67
Pascali et al^29^	16 cases	Yes, if necessary + routine orbicularis myotomy	Endotine Forehead (3.0)	Endotine Ribbon + suture (4/0 PDS)	3.75 ± 1.75	4.95 ± 1.45	0.2 ± 1.8
Badin et al^30^	NA	Yes + routine orbicularis myotomy	No	Fascial fusion (2/0 nylon)	No	2.7 ± NA	3.5 ± NA
Papadopulos et al^14^	1 case	Yes	Percutaneous cortical screw	Percutaneous cortical screw	5.3 ± 1.3	5.6 ± 1.8	5.0 ± 1.5
Jones and Lo^22^	6 cases	Yes	Cortical tunnel + suture (2/0 PDS)	Fascial fusion (3/0 Monocryl)	3.5 ± 3.65	3.4 ± 2.95	2.5 ± 3.35
Massoud and Aboelatta^31^	NA	NA + routine orbicularis myotomy	Group 1: Cortical screw + suture (3/0 Prolene) Group 2: No	Group 1: Fascial fusion (3/0 Vicryl) Group 2: Concentric cable fixation with suture (2/0 Prolene)	No	6.0 ± 1.5	Excluded from evaluation^a^
Perez et al^4^	35 cases	No	No	Endotine Forehead (3.0)	No	1.6 ± NA	No
Gülbitti et al^23^	NA	No	No	Cortical screw + suture (3/0 Ethilon) + Tisseel	3.8 ± 3.4	4.3 ± 3.1	4.4 ± 3.6
Şibar et al^32^	NA	No	No	Prolene mesh + suture (4/0 PDS)	1.97 ± 0.62	2.61 ± 0.89	4.37 ± 0.97

NA, not available; PDS, polydioxanone; SD, standard deviation. ^a^Lateral brow elevation measurements were excluded from the evaluation because they were conducted on an oblique vector.

### Methods for Medial Brow Fixation

Medial brow fixation methods differed across studies. Although several studies did not describe use of any fixation materials, others reported various devices for fixation. The specific methods are summarized in Table 2.

### Methods for Lateral Brow Fixation

Lateral brow fixation methods showed greater variability due to the increased mobility of the lateral brow. Common techniques included fascial fusion and different fixation materials (Table 2).

### Evaluation of Objective Data

**Figure 6.**
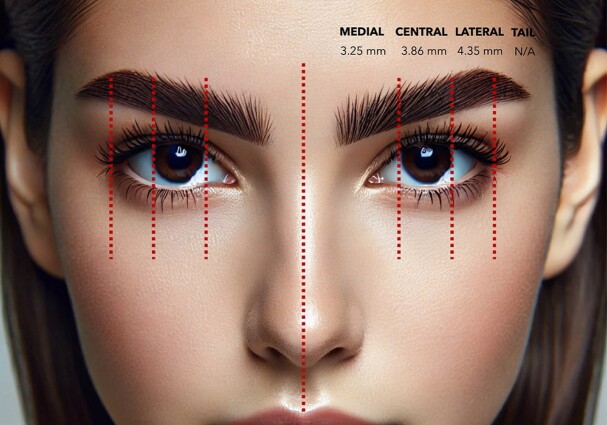
Visual representation of the typical brow elevation outcomes as reported in the included studies. The medial, central, and lateral sections show average elevations of 3.25 mm, 3.86 mm, and 4.35 mm, respectively. The tail region has no specific elevation data available (NA). This image was created with DALL-E, an AI-powered text-to-image model developed by OpenAI (San Francisco, CA).

Most studies provided data on medial, central, and lateral brow elevation, although several did not include measurements of the brow tail. The objective data from each study are summarized in Table 2. According to the meta-analysis results of the available 12 studies, the weighted average brow elevation amounts obtained over the long term from endoscopic brow lifts were as follows: for medial brow: 3.25 mm (2.44-4.06 mm), for central brow: 3.86 mm (2.93-4.8 mm), and for lateral brow: 4.35 mm (3.06-5.64 mm) (Figures 2-4, 6 and Table 2).^4,15,18,24,25,28-34^ Figure 6 was generated with DALL-E (OpenAI, San Francisco, CA), an AI-powered text-to-image model. The authors prompted the DALL-E tool to depict the typical brow elevation outcomes as reported in the included studies, in which the medial, central, and lateral sections showed the average elevations of 3.25 mm, 3.86 mm, and 4.35 mm, respectively. In most of the studies regarding the brow tail (83%), a quantitative evaluation was not performed, therefore a meta-analysis could not be methodologically performed.

## DISCUSSION

Endoscopic brow lift is a frequently preferred method in the surgical treatment of brow ptosis. However, there is no consensus in the literature regarding its long-term effectiveness.^1,19-21,35^ The most frequently criticized and debated aspects of the technique are stabilization problems and recurrent brow ptosis in the long term.^12,21,22^ These problems have led to the development of numerous fixation materials for stabilization today.^19,36^ The etiology of recurrent brow ptosis is complex and can occur in the early or late stages. Early brow ptosis is usually associated with surgical technique errors, while late ptosis is attributed to frontal muscle relaxation, repetitive facial expressions, and gravity.^1,20,37^

Romo III et al have suggested that the chronological relationship between the periosteum's reattachment after fixation and the relaxation of the frontal muscle may play a critical role in recurrent brow ptosis.^35^ If frontal muscle relaxation occurs before the periosteum adheres to its new position after surgery, brow ptosis may develop. Studies in the literature indicate that stable adhesion of the periosteum to its new position occurs within 4 to 12 weeks.^38-40^ Therefore, it can be concluded that the fixation placed should provide stabilization for at least 12 weeks to achieve successful results. It was observed that in 7 of the studies included in the analysis (58.3%), no material was placed for medial brow fixation, and in 10 studies (83.3%), fixation material was placed for lateral brow fixation. This suggests that the need for brow fixation may vary regionally. Studies indicate that myotomy is generally sufficient for the medial region of the brow, suspension is necessary in the central region, and suspension is essential for the tail of the brow.^33,41^ Troilius reported that when comparing the results at 1 year and 5 years after subperiosteal endoscopic brow lift, the brow height progressively increased by an average of +1.9 mm laterally, +2.5 mm centrally, and +2.2 mm medially.^18^ Similarly, Graf observed this effect in the medial brow in the early period and across the entire brow in the late period, regardless of whether fixation was performed. This progressive elevation in the brow is attributed to wound contractile forces, myotomy of depressor muscles, and the functioning of the frontal muscle without the presence of depressor force.^42,43^

Contrary to these findings, most studies report recurrent brow ptosis (especially in the lateral part) after surgery. McKinney and Sweis reported an average loss of 2 to 3 mm in elevation within the first 3 months postoperatively.^44^ In a study of 100 patients, Kim et al found an average loss of 3.26 mm in the lateral brow at 6 months with single cortical tunnel fixation, whereas dual cortical tunnel fixation resulted in a loss of 2.17 mm.^45^ Byrne reported relapses ranging from 0 to 2.4 mm within the first 6 months.^46^ Jones, in 104 cases in which fibrin glue fixation was performed, reported an average relapse of 2.14 mm in the central brow between the early and late postoperative periods.^47^

The increased incidence of ptosis in the lateral brow compared to the medial part can be explained by underlying anatomical differences. These differences include the close relationship between the lateral brow compartment and the temporal fat compartments, its lack of periosteal tissue coverage, decreased frontal muscle activity, and increased depressor muscle activity.^5^ Among the studies included in the meta-analysis, fascial fusion (fixation of the superficial temporal fascia to the deep temporal fascia) was the most commonly performed method for lateral brow fixation (41.6%). Reports indicate that fascial fusion provides reliable and stable results in the long term, while cortical tunnels are recommended in cases of significant brow elevation (>4 mm) and brow asymmetry.^18,25,30,32,48^

Additionally, Angrigiani et al reported in their study that the distal (inferior) portion of the frontal muscle separates from the deep galeal layer and intermingles with the orbicularis oculi muscle. This separation creates 2 distinct fat compartments (gliding planes) between the frontal muscle, deep galeal layer (subgaleal plane), and periosteum at the brow level. The movement of the frontal muscle and, consequently, the elevation of the lateral brow, are achieved through the movement of the distal portion of the muscle toward the middle and the upward movement of the superficial portion over the deep galeal layer. This finding provides significant insights into the use of superficial layers to achieve effective lateral brow elevation in endoscopic brow lifts.^49^ These anatomical characteristics play a crucial role in brow elevation; however, ensuring the longevity of the results requires attention to additional technical considerations. Although endoscopic brow lift relapse is largely related to the fixation method and underlying anatomical differences, an important technical factor that can also contribute to relapse is the inadequate release of all retaining ligaments. A thorough and adequate release of these attachments is critical to achieving a successful and stable outcome.

In addition, focusing solely on the vertical vector during brow lift may not address the natural aging dynamics. Aging often causes inferomedial drooping, and lifting the brow only superiorly may contribute to relapse or create additional strain on the depressor muscles. To achieve more stable and natural results, brow elevation should also consider lateral and superior vectors, aligning the lift with the patient's unique aging pattern. Beyond technical considerations, achieving aesthetically pleasing results also requires careful attention to brow positioning, particularly avoiding excessive medial brow elevation. Excessive medial brow elevation is generally considered inappropriate in most cases, because it often leads to unnatural aesthetic results. This issue, along with the recurrence of lateral ptosis, contributed to the decline of brow lift techniques in the early 2000s. Baker and Chiu emphasized that an ideal brow shape should focus on lateral elevation, avoiding significant medial changes.^22^ Furthermore, Yaremchuk highlighted the challenges of correcting overly elevated medial brows, offering insights into the necessity of brow lift reversal in some cases.^50^ These studies underline the importance of maintaining a balanced brow elevation to achieve aesthetically pleasing outcomes.

In our analysis, we found that endoscopic brow lift typically results in an average elevation of 3.25 mm for the medial brow, 3.86 mm for the central brow, and 4.35 mm for the lateral brow. However, these values may not always align with the clinically desired outcomes. Specifically, a key consideration is that the targeted elevation amounts for the medial and central brow are often lower, with 1 to 2 mm being sufficient, whereas lateral elevation should ideally reach 4 to 5 mm, and the brow tail around 6 to 7 mm.

If the planned average brow elevation for patients exceeds these values, the following methods might be beneficial to consider: additional or multiple fixation methods (such as dual cortical tunnel instead of single or combining cortical tunnel with fascial fusion laterally); combined fixation methods in different tissue planes (such as subperiosteal + subcutaneous cable sutures); or increasing the amount of intraoperative overcorrection (such as +5-8 mm); sharing the advantages and limitations of the technique with the patient or evaluating alternative technique options.

Most of the publications in the literature are case series and lack quantitative data and statistical analysis. In studies with quantitative data, there is heterogeneity in measurement methods (proportional, numerical, or percentage) and vectors (oblique or vertical).

Although these studies were excluded from the evaluation, many of the studies included in the analysis were retrospective in nature and had low levels of evidence. Additionally, simultaneous upper blepharoplasty in some studies, uncertainties regarding patient selection, lack of demographic data, heterogeneity in surgical techniques (such as variations in depressor muscle modifications), the use of different fixation methods, and variability in average follow-up periods (1-6 years) represent the limitations of this analysis.

The effect of simultaneous upper blepharoplasty on brow height is a controversial topic, but the prevailing view in the literature is that it generally does not have a negative impact on brow elevation.^4,24,28,29,51^ The 4- to 5-year results are a more accurate period for assessing long-term permanence. However, the number of studies with follow-up periods longer than 4 years is limited, making meta-analysis impossible. Therefore, to increase the sample size, publications with a follow-up period of more than 1 year were included in the meta-analysis.

In long-term studies, Papadopulos et al reported an average elevation of +5.6 mm in the central brow region over an average follow-up period of 66 months (24-108 months) in 52 patients.^15^ Gülbitti et al reported an average elevation of 3.6 mm in the medial brow, 4.3 mm in the central brow, and 4.4 mm in the lateral brow during an average follow-up period of 6 years (3-11 years) in 47 patients.^25^ In a study by Jones involving 31 patients with an average follow-up of 5.4 years (2.5-10 years), an elevation of 3.5 mm was observed in the medial brow, 3.4 mm in the central brow, and 2.5 mm at the level of the lateral canthus, while the lateral brow tail was found to be only 0.7 mm higher than the preoperative level and remained in nearly the same position as before surgery.^24^ This study by Jones highlights the cause of discussions surrounding relapse in endoscopic brow lifts.

In most of the studies included in the meta-analysis (83%), lateral brow elevation was evaluated, but it was noted that the brow tail was not included in this assessment. Another important point is the visual outcomes of the studies included in the meta-analysis. Postoperative images from all 12 articles evaluated a total of 69 brows.^4,15,18,24,25,28-34^ It was observed that the final peak point of the brow was located medially in 27 brows (39%), centrally in 35 brows (51%), and laterally in only 7 brows (10%). Although the existing studies showed a numerical increase in brow elevation, the aesthetic results were often not ideal. These observations highlight the evolving challenge of achieving ideal aesthetic outcomes, a shift that has been reflected in changing surgical techniques and preferences. Over the past 30 years, brow lift techniques and aesthetic preferences have changed significantly. In the 1990s and early 2000s, brow lifts often aimed for more dramatic and higher positions, which may now be seen as overcorrected. As surgical techniques have evolved, a better understanding of how the brow ages has also emerged.

Eyebrow position changes with age, and different regions of the brow are affected in various ways. Researches show that the medial and central brow tend to rise with age, whereas the lateral brow may remain stable or droop slightly.^52,53^ These changes are influenced by factors such as muscle activity and gravity. Therefore, brow lift techniques should be tailored to account for these aging patterns, aiming for a balanced and natural result.

Current trends favor more subtle, natural-looking elevations, especially in the lateral brow, to avoid an exaggerated appearance. This shift reflects a growing preference for conservative outcomes and underscores the importance of adapting surgical techniques to meet modern expectations. The studies in this review, spanning 1994 to 2024, highlight this evolution in brow lift practices and patient preferences. Maintaining the ideal brow shape has become a key focus in modern brow lift procedures. To achieve this, minimal elevation in the medial brow and an additional 5 to 6 mm of elevation in the tail compared to the medial brow are required. These issues represent some of the most significant challenges in the current literature.

The high I^2^ values observed in the study (93.07%-97.73%) indicate significant heterogeneity, suggesting that the sampled studies were spread across a wide spectrum. This implies that the findings may be influenced by variables such as different fixation methods and patient populations, thereby increasing the generalizability of the results. The presence of heterogeneity reflects the broad scope of the study and indicates that data obtained under different conditions have been brought together.

Furthermore, the results of the publication bias tests showed no significant asymmetry in the funnel plot of the studies. This indicates that the selected studies were evaluated impartially for suitability in the systematic review and meta-analysis. However, the fact that the majority of the included studies were retrospective in nature, with only 1 study being prospectively designed, may impose limitations on the generalizability of the findings.

## CONCLUSIONS

Achieving good stabilization and avoiding recurrent ptosis in the long term are critical factors for success in endoscopic brow lifts. The preservation of brow elevation is an important indicator of stability, and the brow tail is the most critical anatomical region in this regard. There are very few studies in the literature that conduct quantitative analyses on the brow tail, and additional research is needed.

Our study is the first meta-analysis that investigates the average brow elevation amounts over the long term based on existing literature data. The cumulative values obtained in this study may provide guidance in patient selection and may suggest the utilization of additional or combined methods in selected cases. However, prospective, large-sample, randomized controlled studies are needed to better understand long-term efficacy and stability.
